# Artificial intelligence in retinal care: transforming the doctor-patient partnership

**DOI:** 10.1186/s40942-026-00837-y

**Published:** 2026-03-30

**Authors:** Joel Hanhart, Leo Anthony Celi, Eytan Z. Blumenthal, Ronit Almog, Joachim Behar

**Affiliations:** 1 Medical Retina Unit, Department of Ophthalmology, Shaare Zedek Medical Center, The Hebrew University of Jerusalem, Jerusalem, Israel; 2 Laboratory for Computational Physiology, Massachusetts Institute of Technology, Cambridge, MA USA; 3 Division of Pulmonary, Critical Care and Sleep Medicine, Beth Israel Deaconess Medical Center, Boston, MA USA; 4 Harvard Medical School, Boston, MA USA; 5 Department of Ophthalmology, Rambam Health Care Campus, Haifa, Israel; 6 Ruth and Bruce Rappaport Faculty of Medicine, Technion-Israel Institute of Technology, Haifa, Israel; 7 Epidemiology Unit, Rambam Health Care Campus, Haifa, Israel; 8 Faculty of Biomedical Engineering, Technion-IIT, Haifa, Israel; 9 Faculty of Data and Decision Sciences, Technion-IIT, Haifa, Israel

**Keywords:** Artificial intelligence, Diagnostic relationship, Artificial intelligence transformation, Diabetic retinopathy, Age-related macular degeneration, Medical epistemology, Clinical decision-making, Patient-physician partnership

## Abstract

**Background:**

The integration of artificial intelligence into retinal practice represents more than a technological advancement; it constitutes an anthropological shift fundamentally redefining the centuries-old therapeutic partnership between physician and patient.

**Objective:**

To examine how artificial intelligence integration into retinal care transforms the therapeutic partnership, revealing what this transformation illuminates about the nature of medical knowledge itself and identifying frameworks for conscious implementation.

**Methods:**

We conducted a structured narrative review examining AI-based diagnosis and monitoring of diabetic retinopathy and age-related macular degeneration (2018–2025), synthesizing clinical deployment evidence with qualitative implementation studies, adopting an anthropological interpretive stance to examine technology as a mediator of human relationships.

**Results:**

FDA-approved AI systems demonstrate robust diagnostic performance for diabetic retinopathy and age-related macular degeneration. AI integration shifts encounters from examination-based to screen-mediated, clinical reasoning from individual to algorithm-guided, and physicians from diagnosticians to interpreters. Three insights emerge. First, AI reveals that medical practice always combined mechanistic reasoning with pattern recognition, now separated algorithmically. Second, accountability operates asymmetrically: while all stakeholders derive benefits from algorithmic integration, authority over system selection and deployment remains concentrated among vendors and institutions rather than distributed to frontline clinicians or patients. Third, impact diverges along existing stratification: transformation may create access for excluded populations while potentially eroding relationships for those who had comprehensive care, raising fundamental questions about equitable distribution.

**Conclusions:**

The AI transformation of retinal care offers a revealing mirror of medicine’s algorithmic future. Success demands epistemological rigor, robust evaluation competencies and establishing frameworks for shared accountability among the various stakeholders. Our framework maps six fundamental dimensions where synthesis supersedes substitution: expanding algorithmic capabilities while preserving healing relationships, creating access while maintaining continuity. Medicine can embrace algorithmic intelligence while preserving its humanistic core through conscious choices about epistemology, equity, and the character of practice we create.

## Introduction

### The historical evolution of the therapeutic partnership

 The digital transformation of medicine represents more than technological advancement. It constitutes an anthropological shift fundamentally redefining the therapeutic relationship between physician and patient [1, 2]. Understanding this transformation’s magnitude requires examining how this relationship evolved historically [3–5], enabling us to distinguish elements worth preserving from those ready for transformation.

We use “therapeutic partnership” deliberately throughout this discussion, encompassing the full continuum of diagnosis, monitoring, and treatment. This broader conception recognizes what clinicians have long understood: the diagnostic encounter itself constitutes a foundational therapeutic act, establishing the partnership that underpins all subsequent care.

The Western medical tradition saw this partnership evolve through several key phases. The Renaissance witnessed scientific medicine’s emergence, challenging traditional authority through empirical observation [6]. The Enlightenment emphasized clinical observation, which, alongside the Industrial Revolution, gave rise to evidence-based medicine. Clinical decisions for individual patients became increasingly guided by statistical evidence from large populations [7, 8]. 

Between 1950 and 1990, healthcare underwent significant societal evolution, focusing on patient rights, informed consent, and information access [9]. Patient autonomy emerged as cardinal principle. By the late 20th century, the transition from paternalistic models to shared decision-making was well underway [10]. Patients became active participants, protected by laws governing their rights and data protection, while bioethical considerations expanded beyond clinical boundaries [11–13]. 

### The digital rupture: a new epistemological paradigm

Now, barely three decades into the digital age, we face another fundamental transformation, one offering unprecedented clinical opportunity. Artificial intelligence systems autonomously interpret complex retinal imaging at population scale, achieving specialist-level diagnostic accuracy without physician review at point of care. These systems predict disease progression with accuracy exceeding individual clinical judgment and promise to extend screening capabilities to underserved populations worldwide [14–19]. 

Yet this opportunity arrives bearing profound questions. Retinal subspecialty practice proves particularly instructive for examining them [20]. The field’s heavy reliance on imaging modalities makes it ideally suited for AI applications, positioning it at digital medicine’s vanguard [21]. Ophthalmologists have traditionally worked in intimate proximity with patients: shaking hands, exchanging smiles and glances, peering into their eyes through slit lamps while chatting, explaining findings as images remain displayed. This embodied practice now confronts screen-mediated diagnosis, algorithmic pattern recognition, and episodic technology-mediated encounters that fundamentally transform the healing relationship.

What happens to clinical knowledge when expertise shifts from understanding disease mechanisms to interpreting algorithmic outputs? What does it mean when statistical pattern recognition increasingly supplants causal reasoning? How does AI integration affect the structure and quality of the therapeutic partnership? What emerges in retinal practice today may presage broader medical futures. These questions are not rhetorical; their answers will determine whether algorithmic medicine strengthens or diminishes the healing partnership at medicine’s core. This review examines digital transformation through an explicitly anthropological and epistemological lens, exploring how we might navigate this transition thoughtfully.

## Methods

We conducted a structured narrative review (2018–2025) following PRISMA 2020 guidelines adapted for narrative synthesis [22]. We searched PubMed and Google Scholar for (“diabetic retinopathy” OR “age-related macular degeneration”) AND (“artificial intelligence” OR “deep learning” OR “machine learning”) AND (“clinical deployment” OR “FDA approval” OR “implementation”), and for (“therapeutic relationship” OR “doctor-patient relationship”) AND “artificial intelligence” AND (“qualitative” OR “ethnographic” OR “experience”).

We prioritized studies illuminating the anthropological transformation of therapeutic partnerships, focusing on AI performing routine ophthalmologist tasks in retinal imaging interpretation: diagnosis and disease monitoring through fundus photography and optical coherence tomography. Studies required prospective validation, clinical deployment evidence, or regulatory approval. We excluded AI capabilities beyond routine human assessment (age/sex estimation, systemic disease detection, imperceptible OCT biomarkers), surgical applications, treatment selection algorithms, and laboratory-only studies.

We synthesized clinical performance evidence (sensitivity, specificity, AUC, regulatory status, deployment settings, failure modes) with qualitative implementation studies, using Braun and Clarke’s thematic framework [23] and examining technology as mediator of human relationships.

## Results

### Clinical reality: What is already deployed

Understanding the anthropological transformation requires first establishing what AI systems actually accomplish in clinical practice, distinguishing deployed technologies from research promises.

#### Diabetic retinopathy screening: the first FDA-approved autonomous diagnostic AI

Diabetic retinopathy affects one-third of diabetic patients and causes working-age blindness globally [24]. Although systematic screening prevents 95% of vision loss [25], implementation remains limited by ophthalmologist scarcity, patient non-compliance, and physician non-referral.

IDx-DR achieved FDA De Novo approval in April 2018 as the first autonomous diagnostic AI in any medical specialty, analyzing color fundus photographs to detect diabetic retinopathy with 87.2% sensitivity and 90.7% specificity in pivotal trials involving 900 patients [14]. The system has been deployed in primary care sites across the United States, with the manufacturer reporting deployment in over 1,000 clinics [26]. 

“Autonomous” designation means algorithms interpret images without physician review *at point of care*, functioning as population-level screening to identify patients requiring specialist referral rather than rendering final diagnoses. Contractual liability is assumed by the manufacturer rather than clinicians, an innovative but exceptional arrangement [27, 28]. 

EyeArt received FDA clearance in 2020 based on pivotal trial performance (96% sensitivity, 88% specificity for more-than-mild diabetic retinopathy; 92% sensitivity, 94% specificity for vision-threatening disease) [29, 30]. 

Both systems generate binary outputs designed for screening: “referable diabetic retinopathy detected” or “no referable disease.” The ACCESS randomized controlled trial (2024) demonstrated that autonomous AI screening in primary care increased ophthalmology follow-up among diabetic youth by 23% versus standard referral [31, 32]. A Thai national program processing 7,651 examinations achieved 91.4% sensitivity and 95.4% specificity, slightly below development performance attributed to real-world image quality variations [33]. 

However, clinical deployment reveals critical limitations [34]. Ungradable image rates reach 12–43% with non-mydriatic cameras (versus 3.4% with mydriatic photography) [35, 36], primarily due to media opacities, small pupils, and acquisition quality issues [37]. At typical screening prevalences (10–15%), positive predictive values range from 12 to 51%, creating substantial false-positive burdens requiring confirmatory examinations [31]. Performance demonstrates device dependencies; systems validated on specific cameras may degrade with different hardware [38, 39]. Most significantly, autonomous systems are designed to detect specific pathologies under defined imaging conditions. While validation studies demonstrate robust diagnostic accuracy for their target conditions (meta-analysis: sensitivity 87.7%, specificity 90.6%) [34], these evaluations measure primarily diagnostic performance metrics (sensitivity, specificity, and screening efficiency) rather than clinical outcomes such as vision preservation or treatment success. The capacity of these systems to adapt to unusual presentations, integrate contextual clinical factors, or maintain performance across diverse real-world conditions remains incompletely characterized beyond their validated indications [34, 37–39]. 

#### Age-related macular degeneration: decision support systems

Age-related macular degeneration (AMD) afflicts 288 million people worldwide [40]. Several AI systems analyze OCT imaging to detect neovascular AMD and quantify fluid, though most function as physician-assist devices rather than autonomous diagnostics. Altris AI received CE marking in Europe for AMD detection from OCT, with deployment to date primarily in optometry settings [41]. The Google DeepMind/Moorfields system achieves 94% accuracy recommending urgent versus routine referral across over 50 retinal diagnoses including AMD, but lacks FDA clearance [15]. 

Real-world implementation demonstrates specific clinical utility. One retinal practice implementing AI-based AMD risk stratification reported 87% of neovascular conversions occurring in algorithmically-defined high-risk groups, validating predictive accuracy while reducing overall visit volume 12% by extending low-risk patient monitoring intervals [42]. National-scale deployments have confirmed feasibility: China’s CARE system achieved 84.9% diagnostic consistency across 13 fundus diseases including AMD in real-world clinical practice [43, 44]. though prospective validation remains ongoing.

Multimodal integration combining fundus photography, OCT, and genetic risk scores predicts five-year progression to late-stage AMD (AUC 0.82–0.85) [45], though this remains investigational pending prospective validation.

A critical distinction emerges between two modes of AI deployment, each catalyzing distinct transformations:

1. Autonomous screening systems (IDx-DR, EyeArt) operate without physician review at point of care, functioning as population-level screening to identify patients requiring specialist referral. They extend the caring system’s geographic reach to communities lacking retinal expertise. This transformation is epidemiological: democratizing access reshapes who enters therapeutic relationships and where care begins. It values the efficiency and accessibility advantages of the systems, assuming that the clinical outcome benefit for the patients referred to the clinician will be similar to the current practice.
2. Most AMD applications function as decision support systems, requiring clinician review at each step. The epistemological shift is subtle but profound: whereas traditional algorithms detected discrete features (lesion boundaries, optic disc margins) that physicians then interpreted, contemporary AI systems perform this interpretive synthesis themselves. The physician transitions from primary interpreter of imaging data to validator of algorithmic interpretation.

Both paradigms reconceptualize the therapeutic relationship from dyadic (doctor-patient) to triadic (system-doctor-patient). Screening AI reconfigures the geography of care; diagnostic AI reconfigures the nature of expertise. Together, they position the physician as architect of population screening systems and a hermeneutic guide through diagnostic complexity.

These clinical realities set the stage for understanding deeper transformations. Technical capabilities documented above are not merely efficiency improvements but catalysts for fundamental restructuring of how retinal care is delivered, how medical knowledge is conceptualized, and how healing relationships unfold.

### The transformation underway: redefining partnership and knowledge

#### From embodied examination to screen-mediated diagnosis

Traditionally, retinal care embodied medicine’s intimacy: physician and patient together in examination rooms, the physician examining eyes through slit-lamps, hands positioning the patient’s head, verbal exchange explaining findings, collaborative decision-making about treatment. This embodied encounter created bonds transcending information exchange: trust established through physical presence, competence demonstrated through skillful examination, care expressed through attentive touch and listening [46].

Contemporary practice increasingly diverges from this model. In AI-mediated diabetic retinopathy screening, medical assistants acquire fundus photographs using standardized protocols. Software analyzes images autonomously, generating reports without human interpretation. Primary care providers relay results without ophthalmologic expertise. Negative screens schedule next-year imaging; positive screens trigger ophthalmology referrals. The retinal specialist never encounters screen-negative patients (potentially 85–90% of screened populations) and sees positive screens episodically, primarily for treatment rather than longitudinal relationship-building. This model is partially similar to some imaging-based screening programs, like mammography screening, where the radiologist interprets the image, and the screen-positive patients are referred to the domain expert.

For AMD, telemedicine consultations increasingly occur via video platforms: retinologists review OCT images remotely, treatment recommendations are communicated electronically. Physical examination becomes impossible; encounters become transactional rather than relational; the physician never really meets the patient. Direct examination skills, once central to ophthalmologic expertise, risk atrophy as image-based diagnosis dominates.

#### The epistemological challenge: When pattern recognition separates from clinical judgment

This transformation entails more than logistical changes. It makes explicit tensions long implicit in medical practice. Clinical expertise always combined mechanistic understanding with pattern recognition developed through accumulated experience. Medical training emphasizes causal reasoning: why diabetic hyperglycemia damages retinal capillaries, how microaneurysms form, why hemorrhages occur in specific patterns. This pathophysiological knowledge enables contextual judgment and creative problem-solving. Yet experienced clinicians also recognize disease patterns through accumulated exposure, a form of knowledge that often precedes and guides mechanistic reasoning at the bedside. AI systems render this hybrid practice newly explicit through separation. Deep neural networks identify patterns through statistical correlations across millions of images, operating as pure pattern recognition without mechanistic understanding. They detect referable diabetic retinopathy by recognizing pixel configurations statistically associated with that diagnosis in training data, not by understanding vascular pathology. The algorithm cannot explain pathophysiology, cannot reason about presentations outside its training distribution, cannot integrate contextual factors. What distinguishes algorithmic from human expertise is not the presence of pattern recognition but its separation from mechanistic reasoning and contextual judgment. This separation creates practical tensions. When algorithms recommend referable diabetic retinopathy but physicians observe only mild changes, how should clinical decisions proceed? The algorithm identifies population-level statistical patterns derived from extensive training data. The physician brings mechanistic understanding and awareness of individual clinical context. Effective practice requires managing this tension: recognizing when algorithmic pattern detection surpasses human capacity and when contextual judgment should override statistical inference. The gap can be reduced by expanding the training of the models against true clinical value outcome and not just pattern recognition.

Deep neural networks function as black boxes: inputs produce outputs without transparent intermediate reasoning. Explainability techniques (saliency maps, concept-based models incorporating defined features like microaneurysms and hemorrhages) partially address opacity [47]. Physicians trained in pathophysiological reasoning experience epistemic discomfort with statistical pattern recognition operating through mechanisms they cannot directly observe.

This creates an educational challenge. Traditional diagnostic expertise is built through thousands of hours examining retinas, manually identifying microaneurysms, observing hemorrhages and grading severity. This apprenticeship cultivated not merely pattern recognition but mechanistic understanding of what patterns are pathophysiologically significant. If AI automates pattern recognition, what foundational experience remains? Residents relying on AI grades without scrutinizing fundus photographs never develop visual literacy to recognize when algorithmic assessments seem implausible [48]. The critical question becomes: how do we train physicians to evaluate algorithmic outputs through new competencies (validation evidence interpretation, performance metric analysis, failure mode recognition) while maintaining the experiential foundation that builds independent clinical judgment? The challenge is cultivating expertise adequate to algorithmic collaboration: neither blind trust nor dismissive skepticism, but rigorous critical assessment. Like in other innovative revolutions in medicine e.g. personalized medicine based on wide genetic biomarker profiles, the empirical true clinical outcome of different patterns will be part of physicians’ training.

#### Accountability in the algorithmic age: towards a redistributed responsibility?

When AI and clinician disagree, who adjudicates? When errors occur, who bears responsibility? These questions remain inadequately resolved [49, 50]. 

IDx-DR uniquely assumes contractual liability for diagnostic errors, placing responsibility on the manufacturer. This is innovative but exceptional; most AI vendors disclaim liability, placing responsibility on physicians. This creates what has been termed “liability sinks”: professionals held accountable for AI decisions over which they have limited control [50]. Yet this framing as accountability ambiguity obscures a deeper structural inequity. The question is not merely who bears responsibility when algorithms err, but who holds power when they succeed. Frontline clinicians and patients absorb the risks of algorithmic failure while developers and deploying institutions capture efficiency gains and financial returns. This asymmetry reveals AI integration as fundamentally an equity issue, not simply a liability one.

The concrete manifestations are stark. If autonomous diabetic retinopathy screening misses sight-threatening disease, who is liable: the primary care clinician deploying the technology, the AI developer whose algorithm failed, or the ophthalmologist who never saw the patient [51]? If AMD progression occurs sooner than algorithmic prediction indicated, leading to vision loss despite algorithmically extended monitoring intervals, who bears responsibility? In practice, accountability defaults to clinicians despite their limited influence over algorithmic design, training data curation, or deployment protocols.

Safety studies document systematic failure patterns. Errors typically arise from bypassing AI checks through override protocols, database updates not reflecting recent clinical changes, edge cases outside training data distributions, or device heterogeneity where deployment hardware differs from validation specifications [52]. Responsibility attribution remains contested: developers blame inadequate implementation, institutions blame insufficient training, clinicians blame opaque algorithms.

The fundamental issue is structural: in traditional medicine, responsibility clearly resides with the treating physician. With AI, responsibility is distributed across developers (algorithm design), institutions (implementation), and clinicians (final judgment). However, this distribution parallels a distribution of benefits. AI vendors profit from deployment contracts. Healthcare institutions gain efficiency and throughput. Clinicians access enhanced diagnostic capabilities, expanded treatment opportunities, and potentially reduced medicolegal exposure. Yet authority over system selection, design parameters, and deployment protocols remains concentrated among vendors and institutions rather than distributed to frontline clinicians or patients. Decisions about what clinical functions to automate are made by entities with financial incentives for automation, not by those whose therapeutic relationships are restructured. Patients seeking continuity of care navigate screening algorithms without participating in implementation decisions. Communities lacking technical infrastructure face exclusion from both access and governance. This asymmetry reveals that while all stakeholders derive value from algorithmic integration, the distribution of decision-making authority does not reflect the distribution of operational responsibility or clinical impact. No existing framework reconciles these imbalances, creating legal and ethical ambiguity that undermines trust in the therapeutic partnership.

#### The triadic partnership: patient, physician, algorithm

AI transforms the dyadic therapeutic relationship into triadic configuration: patient, physician, algorithm. This restructuring fundamentally alters power dynamics, maintaining trust, and decision-making processes [2].

Patients experience accessibility improvements: screening becomes available in primary care settings, eliminating ophthalmology appointment barriers. This represents genuine advance, particularly for underserved populations. For many, the alternative to AI-mediated screening is not embodied examination but no screening at all. Perceived algorithmic objectivity appeals to many: automated analysis of thousands of patients seems more reliable than individual physician subjective opinion.

Yet those who previously accessed comprehensive care experience meaningful losses. Technology-mediated encounters feel impersonal compared to direct physician examination. Trust becomes ambiguous: when algorithms diagnose, patients seek human reassurance, but physicians themselves may be uncertain about algorithmic reasoning [53]. Most significantly, longitudinal relationships erode. If screening automation means patients only see specialists when positive screens occur, continuous healing relationships dissolve into episodic treatment transactions.

This tension reveals healthcare’s enduring stratification. The direct examination with unhurried consultation and therapeutic continuity that digital transformation threatens was never universally accessible. Well-resourced systems provided longitudinal relationships; under-resourced settings offered episodic encounters; entire populations were excluded from specialist care. AI-mediated screening thus risks relationship erosion for some and access creation for others. The challenge is ensuring that technological benefits and relational costs distribute equitably rather than amplifying existing disparities.

Healthcare professionals report parallel tensions. Efficiency gains are genuine: AI analyzes images orders of magnitude faster than any human. Yet these benefits exact profound costs. The literature documents the “innovation paradox”: professionals simultaneously recognize AI advantages while experiencing implementation burdens that fundamentally challenge core professional values [54]. Time saved on image interpretation is offset by time troubleshooting technology, managing patients anxious about AI findings, and extensive documentation explaining algorithmic involvement. Improved diagnostic consistency comes alongside concerns about clinical reasoning skill erosion, particularly for trainees who may never develop adequate expertise.

Professional identity evolves, though perhaps less radically than initially appeared. Physicians have always interpreted machine outputs, integrating laboratory results, ECG readings, and imaging findings into clinical decisions. What distinguishes contemporary AI is twofold. First, when AI achieves superior sensitivity and specificity, it shifts the diagnostic bottleneck from pattern recognition to decision-making based on algorithmic assessment, reducing rather than augmenting interpretive work. Second, and more fundamentally, AI increasingly detects patterns beyond human perceptual capabilities: identifying glaucoma risk from fundus photographs through vascular features imperceptible to ophthalmologists, predicting systemic disease from retinal imaging. Here, physicians cannot independently verify findings through direct observation. They must trust or reject outputs they cannot evaluate experientially, requiring a genuinely new epistemic stance.

This evolution demands competencies beyond traditional clinical skills: orchestrating algorithmic insights that transcend human pattern recognition, contextual judgment integrating patient-specific factors algorithms cannot access, empathy and communication technology cannot currently provide, creative problem-solving for presentations outside algorithmic training distributions, advocacy ensuring technology serves patient needs rather than efficiency imperatives alone, and system oversight maintaining human accountability when algorithms fail.

These competencies define medicine’s irreducible human core. The challenge is preserving them while integrating algorithmic capabilities. The risk is that efficiency pressures erode time for these human dimensions, reducing medicine to technical transactions.

A parallel systemic risk emerges as automated screening scales beyond its initial validated contexts: extending AI-based retinal image analysis to broader populations based on diagnostic accuracy metrics alone introduces risks of overdiagnosis and overtreatment. When screening reaches lower-prevalence populations, declining positive predictive values generate cascades of confirmatory examinations and interventions that may exceed clinical benefit, a pattern observed across prior screening programs that prioritized technical performance over patient-centered outcomes. Understanding these relational, professional, and systemic transformations is essential, but insufficient. The question becomes: How can retinal practice navigate this transition responsibly, preserving what remains essential while embracing innovation’s genuine benefits?

### Responsible integration: principles and practice

#### Core principles for preserving humanity in the algorithmic age

Six principles emerge from synthesizing clinical evidence with anthropological analysis:

1. Technology as augmentation, not replacement. AI should enhance rather than replace human judgment and relationships. Autonomous systems are appropriate for population-level screening where access barriers otherwise prevent care, but treatment decisions, complex diagnoses, and individualized care require human expertise. The goal is collaborative intelligence: humans and algorithms complementing each other.
2. Transparency and explainability as fundamental rights. Patients and clinicians deserve understanding of how algorithms reach conclusions. Regulatory frameworks should mandate explainability modules for high-risk applications. When black-box algorithms are deployed, their limitations must be acknowledged explicitly. Expanding research that evaluates the clinical impact of AI deployment and the various patterns of AI–physician decision-making and workflows. Such research will provide transparent evidence to guide system improvement, support causal and mechanistic understanding, and inform decisions on whether to continue, modify, or discontinue use. Transparency and alignment with core clinical values will enable AI systems to adapt to new, effective, and safe care, even when such practices fall outside the current standard of care. This transparency will also serve as a foundation for physicians’ learning and trust.
3. Preserving embodied practice. Even with efficient telemedicine, some encounters require physical presence: hands-on examination, facial expression reading, touch conveying care. Balancing virtual efficiency with in-person connection, particularly for anxious newly-diagnosed patients or complex cases requiring nuanced discussion.
4. Accountability frameworks clarifying distributed responsibility. Developers must ensure algorithmic transparency and timely updates; institutions must implement protocols for integration, training, and oversight; clinicians must verify AI outputs against clinical context and document override rationale.
5. Equity as implementation imperative. Digitalization threatens a therapeutic relationship that was never equally distributed. Healthcare systems range from comprehensive universal coverage to severe resource constraints. For populations currently excluded from specialist examination, AI-mediated screening represents access creation rather than relationship erosion. Inequity emerges not from algorithmic screening itself but from unequal access to diagnostic technologies and enabling infrastructure. When AI outperforms average practitioners, denying it to underserved populations perpetuates existing disparities. The critical question is not whether to deploy AI but how to ensure benefits and costs distribute fairly rather than amplifying embedded stratification.
6. Education preserving clinical intelligence. Medical training confronts fundamental pedagogical tensions: collaborating intelligently with AI without becoming dependent, questioning algorithmic outputs critically without dismissing superior statistical evidence, maintaining causal reasoning competencies in contexts increasingly dominated by pattern recognition. These tensions require cultivating new clinical intelligence that integrates the epistemological dialectic we have documented. Medical training must maintain direct examination competencies not only as a backup for algorithmic failure but to develop the conceptual understanding necessary for intelligent AI collaboration. We teach mathematics despite calculators not because calculators fail but to develop numerical reasoning that recognizes implausible results and understands what calculations mean. Similarly, clinical training must maintain examination competencies and pathophysiological reasoning to enable intelligent interpretation of algorithmic outputs and recognition of contexts requiring human judgment. Education cultivates the conceptual understanding that distinguishes physicians from algorithm operators, preparing practitioners who can synthesize mechanistic reasoning with rigorous algorithmic assessment rather than choosing one epistemology over another.

#### A conceptual framework toward practical navigation

Table 1 maps six fundamental dimensions of retinal practice undergoing transformation, synthesizing the anthropological, epistemological, and ethical shifts documented throughout this analysis. Each dimension reveals tensions demanding deliberate epistemological, ethical, and relational navigation. Success requires synthesis rather than substitution: expanding algorithmic diagnostic capabilities while preserving the healing relationship at therapeutic partnerships’ core.

**Table 1:** Dimensions of transformation in the algorithmic therapeutic partnership

Dimension	Traditional Medicine	Algorithmic Transition	Core Tension
Epistemological Foundation	Mechanistic causal understanding of disease pathophysiology	Statistical pattern recognition from training data correlations	Integration without subordination: maintaining mechanistic reasoning while leveraging algorithmic detection
Knowledge Authority	Physician expertise mediates diagnosis and treatment	Distributed across algorithm, physician, and validation evidence	Adjudication when assessments diverge: whose knowledge takes precedence?
Accountability Structure	Physician bears clinical responsibility	Asymmetrically distributed: liability on clinicians, benefits to vendors/institutions	Power concentration masked as distributed responsibility
Access Pattern	Stratified by healthcare system resources	Differentially transformed across contexts	Simultaneous access creation (underserved) and relationship erosion (well-resourced)
Clinical Competence	Pattern recognition through experiential apprenticeship	Dual expertise: mechanistic reasoning + algorithmic evaluation	Cultivating new intelligence without dependency or dismissal
Therapeutic Relationship	Dyadic partnership (patient-physician)	Triadic configuration (patient-physician-algorithm)	Preserving healing dimensions while expanding diagnostic capacity

## Discussion

### Neither regression nor evolution: AI as epistemological mirror

The transformation documented above raises questions extending beyond retinal practice to the foundations of Western medicine itself. For centuries, medical science has pursued causal explanation inherited from Aristotelian frameworks, aspiring to understand disease through mechanistic chains. Why does diabetic retinopathy occur? Because hyperglycemia damages pericytes, weakening capillary walls, producing microaneurysms that hemorrhage and create progressive ischemia, ultimately triggering pathological neovascularization. This mechanistic understanding represents the theoretical ideal of scientific medicine: not merely describing phenomena but explaining their origins and progression.

Clinical reality, however, has always been more complex. The experienced clinician recognizes patterns before invoking mechanisms. Constellations of signs and symptoms suggest diagnoses through accumulated experience, a form of intuitive knowledge that precedes and often substitutes for explicit causal reasoning at the bedside. Clinical expertise marries pattern recognition to mechanistic understanding [55, 56], though medical education privileges the latter as the foundation of scientific legitimacy, perhaps because pattern recognition appears dangerously close to mere empiricism.

Here lies a revealing irony. The Enlightenment narrative portraying medicine as purely mechanistic science is itself mythological. Physicians have always relied substantially on heuristics, clinical judgment, professional authority, and accumulated experience alongside causal reasoning. The idealized image of the clinician deriving every decision from first principles rarely matched bedside reality, where much knowledge remained tacit, transmitted through apprenticeship, refined through pragmatic trial. AI does not mark epistemological rupture so much as it reveals epistemological continuity: scientific medicine was always more empirical and pragmatic than its rhetoric admitted. What changes is not the presence of pattern recognition in medicine but its explicitness, its scale, and its separation from human cognition.

This separation proves consequential. Algorithms identify correlations with unprecedented precision: pixel configurations correlate with outcomes labeled referable diabetic retinopathy in AI training datasets. Yet what distinguishes algorithmic from human pattern recognition is not empiricism versus mechanism but transparency of operation. The algorithm cannot explain pathophysiology, cannot reason about presentations outside its training distribution, cannot integrate contextual factors that modify interpretation. Where human expertise flexibly integrates pattern recognition, mechanistic understanding, and contextual judgment, algorithmic pattern recognition operates as pure statistical correlation, powerful yet epistemologically circumscribed.

The educational challenge this poses is genuine. When pattern recognition automates, will clinicians retain mechanistic understanding necessary to recognize algorithmic failures? The concern is not technological malfunction but epistemological dependency: practitioners operating systems they cannot independently assess when those systems inevitably encounter presentations distant from training data.

Yet al.gorithmic opacity, while real, differs fundamentally from pre-scientific ignorance. Medieval physicians lacked tools to understand willow bark’s mechanism. Contemporary physicians possess robust methods for evaluating algorithmic systems: validation studies quantifying performance, failure mode analysis, demographic bias audits, prospective monitoring. Understanding AI does not require tracing neural network weights any more than prescribing antibiotics requires quantum mechanics. The challenge lies in cultivating new evaluative competencies: interpreting validation evidence, assessing performance metrics contextually, identifying scenarios where algorithms prove unreliable [57, 58]. This represents different understanding than mechanistic reasoning but remains rigorous and empirically grounded, continuous with medicine’s longstanding pragmatic tradition.

The path forward requires integration rather than replacement. Statistical pattern recognition complements rather than supplants causal understanding, each addressing distinct aspects of medical reality. Causal reasoning excels at explaining mechanisms, predicting novel outcomes, guiding therapeutic innovation. Statistical pattern recognition excels at identifying complex associations exceeding human cognitive capacity, detecting subtle patterns invisible to unaided perception. Clinical practice has always required both modalities; AI renders the pattern recognition component explicit, scalable, and separable from human cognition.

Medical education must evolve to prepare physicians for this dual epistemology. Students must still master pathophysiology, understanding why diseases occur and how treatments work, because such knowledge enables reasoning beyond memorized patterns and adaptation to novel presentations. Simultaneously, they must learn when statistical pattern recognition surpasses mechanistic understanding, how to evaluate algorithmic outputs critically, when to trust algorithms and when contextual judgment should override them. This represents not replacement of human expertise by artificial intelligence but cultivation of new forms of human expertise adequate to the algorithmic age: the capacity to collaborate intelligently with pattern recognition systems while preserving the causal reasoning that distinguishes physicians from algorithm operators. Clinical education requires parallel logic, maintaining examination skills and pathophysiological reasoning that enable physicians to interpret algorithmic outputs intelligently, recognize contexts demanding human judgment, and preserve the conceptual understanding separating medical professionals from mere technicians operating sophisticated instruments they cannot question.

#### Ophthalmology’s instructive position

Ophthalmology occupies a unique position in this transformation. Unlike radiologists who already worked primarily through screens, ophthalmologists historically practiced through direct patient examination, maintaining ongoing relationships through chronic disease management. This embodied practice created strong therapeutic bonds, making screen-mediated diagnosis a fundamental rupture rather than incremental evolution.

This disruption proves instructive precisely because of its bounded nature. Retinal imaging represents a delimited domain relying primarily on visual pattern recognition from standardized images. Most medical practice requires integrating patient narratives, social contexts, and systemic variables that current AI cannot synthesize. The therapeutic partnership in comprehensive care cannot be screen-mediated as retinal screening might be. Yet this limitation makes retinal practice an ideal observatory: if ophthalmology can successfully integrate AI while preserving humanistic elements despite direct patient contact traditions, it offers models for other specialties facing similar transitions. Success requires recognizing that technology integration cannot be merely technological; it must be anthropologically informed, ethically deliberate, and grounded in explicit values about what kind of medicine we want to practice [59]. What we accomplish in retinal practice illuminates certain futures while remaining fundamentally bounded; it proves instructive not despite its limits but because of them.

#### Limitations

This review has limitations. Qualitative evidence derives primarily from general digital transformation studies rather than retinal-specific anthropological research, which remains sparse. Our narrative synthesis without meta-analysis reflects heterogeneity precluding quantitative pooling. Longitudinal studies would illuminate partnership evolution our cross-sectional synthesis cannot capture.

Most fundamentally, our analysis adopts a physician-centered perspective. Patients appear primarily as recipients of transformed care rather than active participants shaping implementation. Community perspectives on digital health equity and infrastructure access remain absent. This reflects both available literature and our own positionality as clinicians and researchers. Future work should center patient and community voices, investigating what aspects of care they value most, how they experience algorithmic versus human assessment, and what trade-offs they find acceptable. Participatory methods engaging patients as co-investigators would address this gap. Ironically, the triadic partnership we document should temper precisely this physician-centered stance: when the algorithm emerges as third partner, both patient and clinician face comparable challenges understanding and negotiating with statistical pattern recognition that transcends either’s individual perspective, inviting humility from all participants rather than professional privilege.

Additionally, our analysis does not address how demographic variables shape therapeutic partnerships and their digital transformation. Gender dynamics in physician-patient relationships, algorithmic performance variation across racial and ethnic groups, and how socioeconomic status and geography determine access to embodied versus mediated care represent critical dimensions beyond this review’s scope. Future research must confront these intersecting dimensions directly, especially as algorithmic medicine may amplify rather than ameliorate longstanding healthcare inequities.

## Conclusions

Digital transformation in retinal practice delivers genuine benefits: expanded access to underserved populations, improved diagnostic consistency, enhanced efficiency. Realizing this potential requires conscious redefinition of the therapeutic partnership itself, which becomes necessarily triadic, involving patient, physician, and algorithm.

The transformation is here. We cannot afford paralysis. The question is whether we shape it deliberately or allow it to shape us by default. Clinical practice has always combined pattern recognition with causal reasoning, though medical education emphasizes mechanistic understanding as the foundation of scientific legitimacy. AI renders this duality explicit and demands integration. Statistical pattern recognition increasingly supplements causal reasoning in daily practice, raising fundamental questions: Are we educating physicians who lack competencies in evaluating algorithmic validation evidence, performance metrics, and failure modes? Or are we evolving toward intentional dual epistemology that integrates mechanistic reasoning with rigorous statistical assessment, cultivating new forms of understanding adequate to algorithmic medicine? The answer depends on choices we make now about medical education, professional identity, and what expertise means in the algorithmic age.

We must preserve what remains irreplaceably human: empathy recognizing patients as whole persons rather than datasets, contextual judgment considering unique circumstances, creative problem-solving for presentations outside training distributions, advocacy ensuring technology serves patients rather than efficiency metrics, and accountability when algorithms fail. These capacities cannot be delegated to pattern recognition systems however sophisticated.

The retinal specialist’s role transforms fundamentally. No longer primarily diagnostician, as algorithms handle routine pattern recognition, the specialist becomes interpreter explaining algorithmic outputs within clinical context, orchestrator coordinating care across platforms, educator helping patients navigate self-monitoring technologies, and guardian ensuring technology enhances rather than erodes healing relationships.

Digital transformation’s impact varies profoundly across healthcare contexts. For populations previously excluded from specialist examination, AI-mediated screening represents access creation rather than relationship erosion. For those who accessed comprehensive care, technology-mediated encounters diminish aspects of healing that require physical presence and longitudinal relationships. The embodied practice we risk losing was never universally accessible. This stratification existed before digitalization; the challenge is ensuring technological benefits distribute equitably rather than amplifying existing disparities.

Three domains require immediate action. Training programs must prepare physicians for dual epistemology: maintaining mechanistic understanding while developing competencies in evaluating algorithmic performance through validation studies, failure mode analysis, and demographic bias auditing. Regulatory frameworks must clarify accountability when autonomous systems fail, addressing power asymmetries where those capturing benefits rarely bear proportionate responsibility for failures. Healthcare systems must allocate resources not merely for technology acquisition but for protected time enabling physicians to fulfill irreducible human functions.

What is at stake is medicine’s soul itself. The transformation’s magnitude rivals previous medical revolutions. Whether it enhances or diminishes medicine’s healing mission depends on choices we make now, deliberately and urgently. The question is not whether transformation occurs but what kind of medicine emerges: one where algorithms serve healing relationships while expanding access equitably, or one where efficiency imperatives erode therapeutic bonds without ensuring equity for all. We must act thoughtfully to redefine the therapeutic partnership for the AI algorithmic age, maintaining vigilant awareness of epistemological shifts and their implications for what it means to know, to heal, and to care for patients as whole human beings. 

## Data Availability

No datasets were generated or analysed during the current study.

## Abbreviations

AI: Artificial Intelligence
AMD: Age-related Macular Degeneration
AUC: Area Under the Curve
CARE: Comprehensive Artificial Intelligence Retinal Expert
CE: Conformité Européenne (European Conformity marking)
DR: Diabetic Retinopathy
ECG: Electrocardiogram
FDA: Food and Drug Administration
OCT: Optical Coherence Tomography
PRISMA: Preferred Reporting Items for Systematic Reviews and Meta-Analyses
